# Transregional movement of multidrug-resistant tuberculosis in north China: an underlying threat to tuberculosis control

**DOI:** 10.1038/srep29727

**Published:** 2016-07-14

**Authors:** Jun An, Mengqiu Gao, Naihui Chu, Hairong Huang, Yu Pang, Liang Li

**Affiliations:** 1 Beijing Chest Hospital, Capital Medical University, Beijing, China; 2 Beijing Tuberculosis and Thoracic Tumor Research Institute, Beijing, China; 3 National Center for Tuberculosis Control and Prevention, Chinese Center for Disease Control and Prevention, Beijing, China; 4 Administration Office, Clinical Center on Tuberculosis, China CDC, Beijing, China

## Abstract

Due to unbalanced distribution of health care resource in China, tuberculosis patients, especially multidrug-resistant tuberculosis (MDR-TB), prefer to suffer transregional movement for seeking better health care service in the first-tier cities. Here, we performed a study on the current status of transregional movement of tuberculosis in northern China by reviewing the medical record of TB patients hospitalized in Beijing Chest Hospital from 2011 to 2015. From 2011 to 2015, the proportion of non-Beijing in-patients had increased from 55.0% (996/1810) to 67.2% (1860/2769). In addition, we found that the rate of re-treated among non-Beijing group was significantly higher than that among Beijing-group. Compared with the proportion of pulmonary TB patients from non-Beijing, there were more extra-pulmonary TB patients from non-Beijing. In addition, 10.5% (164/1568) of Beijing in-patients and 26.5% (491/1858) of non-Beijing in-patients had MDR tuberculosis, and statistical analysis revealed that there was significantly higher proportion of MDR cases among non-Beijing group than Beijing group. In conclusion, our data demonstrate that an increasing proportion of TB patients from northern China seek health care in Beijing. In view of higher prevalence of MDR-TB cases among these patients, the transregional movement of MDR-TB will lead to ongoing MDR TB transmission in the community.

Tuberculosis (TB), caused by *Mycobacterium tuberculosis* complex (MTBC), is one of most serious infectious diseases in China^1^,^2^. Despite the great progress that has been made in recent years, China had an estimated 0.93 million new tuberculosis cases in 2015, accounting for 10% of global tuberculosis burden^3^. The most threatening obstacle for TB control and prevention strategy in China is a serious epidemic of multidrug-resistant TB (MDR-TB), defined as the strains resistant to at least isoniazid (INH) and rifampicin (RIF)^4^,^5^,^6^. According to a national drug resistance survey conducted in China, 5.7% of new TB patients and 25.6% of previously treated patients had MDR-TB^4^,^7^. This far surpasses the global average where approximately 3.5% of new cases and 20.5% of the retreated cases are MDR^8^.

In recent years, as a result of rapid socioeconomic growth, patients, also including TB patients, are able to enjoy a better quality of health care in China^9^, whereas the distribution of health care service is unbalanced between different regions^10^. The best health care service is always centralized in the first-tier cities like Beijing and Shanghai^11^. The TB patients from resource-limited setting in China, especially drug-resistant patients, may not timely access to rapid case detection and appropriate treatment, thereby leading to the unsatisfactory treatment outcome^12^. More importantly, these patients with poor clinical outcome prefer to carry out transregional movement for seeking better health care service in the first-tier cities. The transmission of MDR-TB patients will cause the community transmission in China. Although this is a serious obstacle associated with TB control and prevention, there is no systematic report on the current status of transregional movement of tuberculosis and MDR-TB in China.

Beijing Chest Hospital is one of the best TB specialized hospitals in China^13^. In addition to provide clinical service for residents in Beijing, a large number of TB patients from all over China, especially from north China, seek health care in this hospital. In this study, we reviewed the medical record of TB patients hospitalized in Beijing Chest Hospital from 2011 to 2015. Our aim was to analyze the proportion of in-patients contributing to the transregional movement in Beijing Chest hospital, and the factors associated with this movement.

## Results

### Number of patients

**Figure 1:**
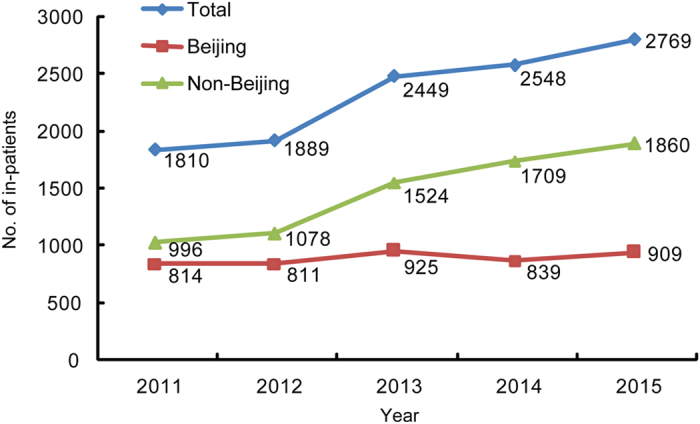
Trends of Beijing and non-Beijing TB in-patients seeking health care in Beijing Chest Hospital.

A total number of TB in-patients treated in Beijing Chest Hospital increased from 2011 to 2015 (Fig. 1). In comparison to the 2011 baseline, this represented increases of 4.4%, 35.3%, 40.8% and 53.0% for 2012, 2013, 2014 and 2015, respectively. From 2011–2015, Beijing resident TB inpatient number remains the same or slightly increases annually from 814 to 909, but non-Beijing inpatient number almost doubled from 996 to 1860. Further analysis confirmed that the increasing trend was significantly different between Beijing and non-Beijing groups over the past five years (Chi-square trend 107.68, *P* < 0.001).

### Geographic origins of patients

**Figure 2:**
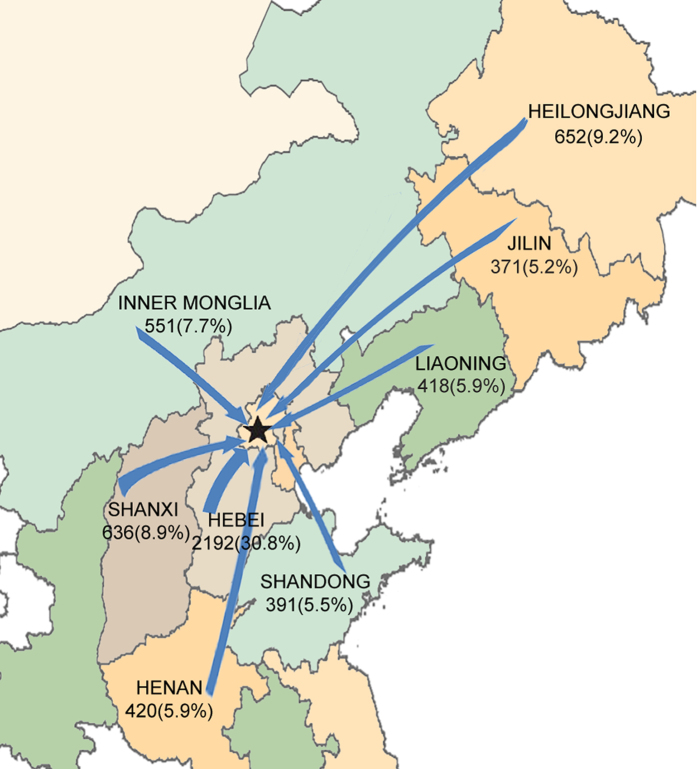
Maps of transregional movement of tuberculosis in-patients from surrounding regions to Beijing. The number annotated in the figure represents the number and the proportion of TB in-patients from each province between 2011 and 2015. The background map was created using Matlab 7.0 software (The MathWorks, MA, USA, www.mathworks.com/products/matlab), Adobe Photoshop 6.0 (Adobe Systems Inc., CA, USA, http://www.adobe.com/cn/products/photoshop.html) and Adobe Illustrator CS4 (Adobe Systems Inc., CA, USA, http://www.adobe.com/products/illustrator.html).

We further analyzed the geographic origins of TB in-patients according to their residence between 2011 and 2015. A total of 7117 in-patients were classified as non-Beijing group. As shown in Fig. 2, of these in-patients, 79.1% (5631/7117) came from the eight surrounding provinces of Beijing, including Hebei, Shanxi, Inner Mongolia, Henan, Shandong, Liaoning, Jilin and Heilongjiang. Hebei Province contributed the largest number of transregional in-patients, accounting for 30.8% (2192/7117) of all non-Beijing in-patients, which might be associated with the shortest distance from Beijing. In addition, Heilongjiang, a northern east province located more than 1200 kilometers away from Beijing, ranked second in the survey, and 9.2% (652/7117) of non-Beijing in-patients came from this province.

### Factors associated with transregional movement of TB patients

A classification of TB in-patients belonged to Beijing residents, stratified according to gender, age, medical history, and clinical diagnosis was shown in Table 1. We found that there was higher rate of women belonging to non-Beijing residents when compared with that belonging to Beijing residents [OR (95% CI): 1.71(1.57–1.85)]. Statistical analysis revealed that the percentage of in-patients exhibited different distribution pattern between Beijing and non-Beijing group according to age groups. The percentage of non-Beijing in-patients was higher among young aged group [aged under 25 years OR (95% CI): 1.71(1.49–1.95)], whereas the percentage of non-Beijing in-patients aged 45–65 years [OR (95% CI): 0.29(0.26–0.32)] and aged >65 years [OR (95% CI): 0.11(0.10–0.13)] was significant lower. In addition, we found that the rate of re-treated among non-Beijing group was significantly higher than that among Beijing-group [OR (95% CI): 1.22(1.10–1.36)], indicating more re-treated TB cases contribute to transregional movement for seeking health care. Compared with the proportion of pulmonary TB patients from non-Beijing, there were more extra-pulmonary TB patients from non-Beijing, including tuberculous meningitis [OR (95% CI): 2.65(2.05–3.42)], skeletal tuberculosis [OR (95% CI): 3.70(2.97–4.61)] and others [OR (95% CI): 1.77(1.42–2.20)].

### Comparison of drug susceptibility profiles between Beijing and non-Beijing in-patients

**Figure 3:**
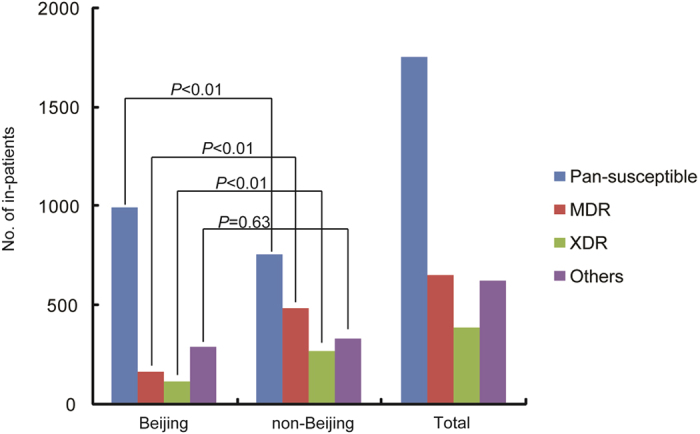
Comparison of drug susceptibility profiles between Beijing and non-Beijing TB in-patients.

Drug susceptibility profiles of *M. tuberculosis* isolates from Beijing or non-Beijing in-patients were compared. Out of 11465 TB in-patients from Beijing Chest Hospital, the DST results of 3424 in-patients were available for our analysis, including 1568 (45.8%) from Beijing in-patients and 1858 (54.3%) from non-Beijing patients. Overall, a total of 655 in-patients were identified as MDR tuberculosis by conventional DST. As shown in Fig. 3, 10.5% (164/1568) of Beijing in-patients and 26.5% (491/1858) of non-Beijing in-patients had MDR tuberculosis, and statistical analysis revealed that there was significantly higher proportion of MDR cases among non-Beijing group than Beijing group (Chi square 140.57, *P* < 0.01). In addition, there were 116 (7.4%, 116/1568) and 272 (14.7%, 272/1858) XDR cases in Beijing and non-Beijing group, respectively. Similarly, non-Beijing in-patients provided higher proportion in XDR tuberculosis than Beijing in-patients (Chi square 44.55, *P* < 0.01). In contrast, we observed that the percentage of pan-susceptible *M. tuberculosis* isolates of non-Beijing group was significantly lower than that of Beijing group (40.8% vs. 63.5%, Chi square 173.99, *P* < 0.01).

## Discussion

China has achieved an important global milestone toward tuberculosis elimination: to reduce the prevalence of smear-positive tuberculosis by 2015, while the epidemic of drug-resistant TB, especially MDR-TB, threaten tuberculosis control efforts in China^1^. In the present study, we firstly described a large-scale transregional movement of multidrug-resistant tuberculosis in north China, which provide several important hints for national tuberculosis programmes. The most important reason for this movement is the unbalanced distribution of medical resources in China. Although there are several TB specialized hospitals in each province, obvious gaps exist from laboratory diagnosis to clinical treatment between these hospitals and Beijing Chest Hospitals. Hence, a proportion of TB patients with unfavorable clinical outcome in the local hospitals are prone to seek health service in Beijing, which may be responsible for higher proportion of re-treated and MDR-TB cases in non-Beijing group.

Notably, we found that the proportion of transregional tuberculosis in-patients for health care in Beijing was significantly increased from 2011 to 2015. There were several potential reasons for this growing trend. First, due to sufficient fund support and appropriate control strategies, Beijing is one of the provinces with low TB burden in China. In 2015, the incidence of TB is 32 per 100 000 population in Beijing, which is less than half of national average level (68 per 100 000 population)^14^. The low epidemic of TB in Beijing is the most important reason for the lower percentage of TB in-patients. Second, benefit from rapid economic development in the past decades, the average income level has significantly increased in China^15^. The improved economic status allows the non-Beijing patients to have an opportunity of seeking better health care in Beijing after a long journey. Due to the implementation of the DOTS strategy in the public health system, China more than halved its TB prevalence over the past 20 years^1^. Unfortunately, progress in the management of MDR-TB has been unsatisfactory, and approximately 52,000 people become ill with MDR TB in China each year. When compared with drug-susceptible TB, who can be cure in >95% with 6 month of standardized treatment, treatment for MDR-TB always requires prolonged use of toxic and expensive second-line anti-TB drugs, and succeeds in about 50% of cases^6^. As a result of these problems, MDR-TB is a major concern for TB control programs in China^4^,^16^. The emergence of MDR-TB is considered as a man-made phenomena due to inadequate treatment of TB and poor infection control in health healthcare facilities and congregate settings^17^. Considering most of non-Beijing patients prefer to take public transportation rather than personal vehicles, the large-scale transregional movement of MDR-TB patients will probably play a critical role in the primary transmission of MDR-TB^18^. Our findings highlight the urgent need to address the challenge of the potential transmission from patient movement. In order to solve this problem, the health inequity remains a major concern under the current situation in China. In recent years, under the support from National Health and Family Planning Commission of China, a nationwide cooperation system has been set up among the TB specialized hospitals, which allows that the TB patients in the resource-limited settings, especially the refractory patients, are able to enjoy better health care from the top-level hospitals. However, more needs to be done, including providing quality MDR-TB service in each city in China and issuing regulations to limit the MDR-TB patient’s movement via public transportation system.

Extrapulmonary TB receives less attention in international TB control strategies, while it contributes significantly to TB^19^,^20^. In this study, we observed that the extrapulmanory TB accounted 12.2% of all TB in-patients, which is in consistent with previous description from countries with high TB incidence (13.2%)^20^. In addition, our results demonstrated that there were significantly higher rate of extrapulmonary TB from non-Beijing group compared with Beijing group (15.7% vs. 6.6%), indicating that the diagnosis and treatment of extrapulmonary TB is challenging in the TB specialized hospitals from non-Beijing regions. On one hand, due to the relative low prevalence of extrapulmonary TB in China and a variety of symptoms that mimic symptoms of other pathologies, the clinicians may have little experience in diagnosing extrapulmonary TB, thereby leading to diagnostic delays or even missed diagnoses^21^. On the other hand, the clinicians also pose a further challenge in treatment of extrapulmonary TB. In China, no guideline for the treatment of extrapulmonary TB has been established till now. The clinical treatment of extrapulmonary TB mainly relies on the experience of clinicians. Therefore, our findings highlight that there are increasing demands for improving the diagnosis and treatment of extrapulmonary TB in China, especially in the resource-limited regions.

There were several obvious limitations in this study. First, this study included only TB in-patients rather than all patients seeking health care in Beijing Chest Hospital, which may result in sample bias. However, considering out-patients always had no medical record in the hospital, the data from out-patients are invalid for our analysis. Second, because these in-patients only received no more than two month anti-TB treatment, there were no further follow-up results to monitor the clinical outcomes. Third, the comorbidities of in-patients were not included in this study. Nevertheless, our study firstly describes the transregional movement of MDR-TB in north China, which will provide important guidance for formulating the appropriate control strategies for TB control in China.

In conclusion, our data demonstrate that an increasing proportion of TB patients from northern China seek health care in Beijing. In view of higher prevalence of MDR-TB cases among these patients, the transregional movement of MDR-TB will lead to ongoing MDR TB transmission in the community. In addition, the higher rate of extrapulmonary TB from non-Beijing group is observed when compared with Beijing group, indicating that the diagnosis and treatment of extrapulmonary TB is challenging in the TB specialized hospitals from non-Beijing regions. Our findings highlight an underlying threat to the prevention of MDR-TB in China, which will provide important guidance for formulating the appropriate control strategies for TB control in China.

## Methods

### In-patients

We retrospectively reviewed medical records of all in-patients diagnosed as tuberculosis between 2011 and 2015. TB patients met the national criteria for the diagnosis of tuberculosis^22^. A variety of social-demographic data was collected from medical records including gender, age and place of residence. The patients who were residence of Beijing were classified as Beijing group, while those who sought health care from other provinces were classified as non-Beijing group. The patients belonging to non-Beijing group were further divided according to their places of residence. In addition, we recorded the clinical diagnosis made by clinicians, including pulmonary tuberculosis, tuberculous meningitis, skeletal tuberculosis and others.

### Drug susceptibility testing

The drug susceptibility testing was performed with absolute concentration method on Lowenstein-Jensen media containing the corresponding anti-TB drugs according to the guideline of World Health Organization (WHO)^23^. A total of twelve drugs were used to perform conventional DST, and the concentration of nine anti-TB drugs followed the description as previously reported: rifampcin (RIF), 40 mg/ml; isonizid (INH), 0.2 mg/ml; streptomycin (SM), 10 mg/ml; ethambutol (EMB), 2 mg/ml; kanamycin (KAN), 30 mg/ml; capreomycin (CAP), 40 mg/ml; amikacin (AMK), 30 mg/ml; ofloxacin (OFLX), 2 mg/ml; and levofloxacin (LFX), 2 mg/ml^13^. In addition, media containing paranitrobenzoic acid (PNB, 500 mg/ml) and thiophen-2-carboxylic acid hydrazide (TCH, 5 mg/ml) was parallelly tested to perform Mycobacterium species identification. Pan-susceptible TB was defined as TB that was susceptible to all drugs tested. In addition, multidrug-resistant TB (MDR-TB) was defined as TB that was resistant to the two most effective first-line drugs, isoniazid and rifampin, while extensively drug-resistant TB (XDR-TB) was defined as MDR-TB that was additionally resistant to any fluoroquinolone and one of three injectable second-line drugs (KAN, AMK and CAP).

### Statistical analysis

Variables were tested by chi-square test to compare the distribution of demographic, clinical diagnosis, and drug susceptibility profiles between Beijing residents and non-Beijing residents. In addition, chi-square test for trend was used to test for trends of in-patients over the years. For statistical analysis, SPSS version 15.0 (SPSS Inc., Chicago, IL) was used. The difference was considered as significant if *P* value was less than 0.05.

### Ethics Statement

The protocols applied in this study were approved by the Ethics Committee of the Beijing Chest Hospital, affiliated to Capital Medical University.

## Additional Information

**How to cite this article**: An, J. *et al*. Transregional movement of multidrug-resistant tuberculosis in north China: an underlying threat to tuberculosis control. *Sci. Rep.* **6**, 29727; doi: 10.1038/srep29727 (2016).

## Figures and Tables

**Table 1:** Demographic and clinical characteristics of in-patients enrolled in this study.

Characteristic	No. of in-patients (%)			OR (95% CI^a^	Chi square	*P* value
	Total (N = 11465)	Beijing (N = 4348)	Non-Beijing (N = 7117)			
Gender						
Male	7567(66.0)	3184(73.2)	4383(61.6)	1.00(Ref.)	—	—
Female	3898(34.0)	1164(26.8)	2734(38.4)	1.71(1.57–1.85)	165.23	<0.01
Age group (years)						
<25	2640(23.0)	387(8.9)	2253(31.7)	1.71(1.49–1.95)	61.10	<0.01
25–44	3354(29.3)	760(17.5)	2594(36.4)	1.00(Ref.)	—	—
45–65	3392(29.6)	1699(39.1)	1693(23.8)	0.29(0.26–0.32)	547.74	<0.01
>65	2079(18.1)	1502(34.5)	577(8.1)	0.11(0.10–0.13)	1298.67	<0.01
Medical history						
New case	9576(83.5)	3705(85.2)	5871(82.5)	1.00(Ref.)	—	—
Retreatment	1889(16.5)	643(14.8)	1246(17.5)	1.22(1.10–1.36)	14.50	<0.01
Diagnosis						
Pulmonary TB	10661(93.0)	4063(93.4)	5998(84.3)	1.00(Ref.)	—	—
Tuberculous meningitis	373(3.3)	76(1.7)	297(4.2)	2.65(2.05–3.42)	60.16	<0.01
Skeletal tuberculosis	627(5.5)	97(2.2)	530(7.4)	3.70(2.97–4.61)	154.10	<0.01
Others	404(3.5)	112(2.6)	292(4.1)	1.77(1.42–2.20)	25.97	<0.01

^a^OR: odds ratio; CI: confidence interval.
